# *Pneumocystis jirovecii* in Spanish Patients With Heart Failure

**DOI:** 10.3389/fpubh.2019.00289

**Published:** 2019-10-09

**Authors:** Izarbe Merino-Casallo, Vicente Friaza, Sebastián Menao, José María Domingo, Susana Olivera, Enrique J. Calderón, Miguel Ángel Torralba

**Affiliations:** ^1^Department of Internal Medicine, “Lozano Blesa” University Hospital, Zaragoza, Spain; ^2^Instituto de Investigación Sanitaria Aragón, Zaragoza, Spain; ^3^Instituto de Biomedicina de Sevilla, Hospital Universitario Virgen del Rocío, Universidad de Sevilla, Consejo Superior de Investigaciones Científicas, Seville, Spain; ^4^Centro de Investigación Biomédica en Red de Epidemiología y Salud Pública (CIBERESP), Hospital Universitario Virgen del Rocío, Seville, Spain; ^5^Department of Biochemistry, “Lozano Blesa” University Hospital, Zaragoza, Spain; ^6^Bank of Blood and Tissues of Aragón, Zaragoza, Spain

**Keywords:** *Pneumocystis*, heart failure, epidemiology, pathophysiology, general population, Spain

## Abstract

**Objective:** *Pneumocystis* colonization is frequent in patients with chronic obstructive pulmonary disease (COPD) producing local and systemic inflammation. Heart failure is also a common comorbidity among patients with COPD. Heart failure is a chronic, frequent, and disabling condition with high morbidity and mortality, but with a modifiable course where endothelial dysfunction and pulmonary arterial hypertension have great importance. Animal models have shown that *Pneumocystis* infection can cause relevant functionally changes in vascular responses in the lung, promoting the development of pulmonary hypertension. *Pneumocystis* colonization could be a hidden cause of worsening heart failure through it capacity to induce inflammatory response with subsequent endothelial dysfunction and pulmonary hypertension. The aim of the present study was to investigate the prevalence of *Pneumocystis jirovecii* colonization in heart failure patients and its possible association with reduced or preserved ejection fraction.

**Methods:** A cross-sectional study was carried out including 36 heart failure patients and 36 control cases. Identification of *P. jirovecii* colonization was performed by means of molecular techniques in oropharyngeal washing.

**Results:** *Pneumocystis*-DNA was identified in oropharyngeal washing in 1 (2.7%) of 36 heart failure patients and in 3 (8.3%) of 36 controls.

**Conclusions:** *Pneumocystis* colonization does not seem to have a role in the pathophysiology of heart failure.

## Introduction

Heart failure (HF) is a progressive disease with high morbidity and mortality that affects about 1–2% of the population in developed countries, with an incidence of 5–10 cases per 1,000 persons-year (1). Inability of the heart to maintain sufficient output of blood for the body's demands is the main characteristic of HF causing frequent hospitalizations due to breathing difficulties and fluid overload, risk of deadly arrhythmias, impaired quality of life, and high mortality despite the fact that therapy has developed significantly over the past decades improving outcomes (2, 3). A different approach and improved preventive and therapeutic tools are thus urgently needed.

In animal models, it has been shown that the immune response to an infectious agent, such as *Pneumocystis*, can lead to later development pulmonary hypertension (PH) that persists after the pathogen has been cleared (4, 5). In this model of *Pneumocystis*-associated PH there are a substantial enhancement of the vasoconstrictor response to endothelin-1 and marked reduction in production of adrenomedullin an important vasodilator (6).

PH is a common hemodynamic complication of heart failure. Interest in left-sided PH has increased in recent years as its development and consequences for the right heart are now seen as cornerstone abnormalities that begin in the early stages of the disease and bear unfavorable prognostic insights (7, 8).

*Pneumocystis* colonization is frequent in patients with chronic obstructive pulmonary disease (COPD) in which produces local and systemic inflammation (9, 10). Heart failure is also a common comorbidity among patients with COPD (11). Based on this evidence it can be considered that *Pneumocystis* colonization could be a hidden cause of worsening heart failure through it capacity to induce inflammatory response with subsequent endothelial dysfunction and PH.

The aim of the present study was to investigate the prevalence of *P. jirovecii* colonization in heart failure patients and its possible association with reduced or preserved ejection fraction.

## Patients and Methods

A Cross-sectional study was carried out between February and June 2016. Thirty-six non-HIV patients between 41 and 89 years old with HF diagnosis, echocardiogram and N-terminal pro-brain natriuretic peptide (NT-proBNP) determined in the last 6 months were included. Eighteen were inpatients and 18 were outpatients from the Internal Medicine or Cardiology Department. In the same time, 36 healthy individuals from the Blood and Tissue Bank of Aragon were included as controls. All participants completed written informed consent and, according to our hospital's regulations, the procedure for requesting and authorizing research studies was completed.

Every individual underwent a clinical and biological examination using a standardized protocol, and biological samples were collected for analysis. Oropharyngeal washing, a simple-non-invasive sample collection method consisting of gargling with 10 ml of normal saline solution for 1 min, was used in all cases to identify *Pneumocystis* colonization by mean of 2-step protocol for a nested-PCR assay that amplifies a portion of the gene encoding the mitochondrial large-subunit (mt LSU) ribosomal RNA (rRNA) (12, 13). Briefly, DNA from *P. jirovecii* was extracted using a commercial kit (Qiagen). During the first round of amplification, the external primers pAZ102-E and pAZ102-H were used. This yielded a 346–base pair fragment. The second round of amplification used the internal primers pAZ102-X and pAZ102-Y and yielded a 260–base pair product. Both rounds of PCR comprised 35 amplification cycles. Amplicons were analyzed by electrophoresis on a 1.5% agarose gel containing ethidium bromide, and the bands were visualized by UV (12). To prevent contamination, pipettes with filters were used in all manipulations. DNA extraction and preparation of the reaction mixture were performed in two different rooms, using separate laminar-flow hoods. The PCR procedure and analysis of PCR products were performed in another room. Control samples were run simultaneously with oropharyngeal washing samples. Positive control samples were bronchoalveolar lavage specimens from patients with *Pneumocystis* pneumonia; negative control samples were autoclaved water in the PCR mixture in the absence of the DNA template controls.

SPSS 20.0 was used for analysis and significance set at *p* < 0.05. Applying the Kolmogorov–Smirnov test, variables that presented a deviation from a normal distribution were determined. Univariate analyses were employed to compare clinical characteristics between groups, using either the Mann-Whitney *U*-test for qualitative variables or the Student's *T*-test as a parametric test for the quantitative variables. The analysis of the qualitative variables that adjusted to normality was carried out using Chi^2^. Spearman's correlation coefficient analyzed the association between quantitative variables. Finally, the Kruskal–Wallis test was used for qualitative non-dichotomous variables.

## Results

Heart failure patients were 70.9 ± 7.5 years old (range 41–89) and 18 (50%) of them were female. Half of cases had preserved ejection fraction with the same distribution between male and female. Almost half of the patients studied had a single previous income. NT-proBNP levels were not related with hospital admission, nor to the gender. However, those patients with reduced ejection fraction had higher level of NT-proBNP (3733.61 ± 2845.44 pg/ml) than those with preserved ejection fraction (2583.72 ± 1933.9 pg/ml) (*p* = 0.165).

Most of the patients admitted were in functional class III (NYHA) by the time of the study, while the outpatients had functional class I. Characteristics of patients with heart failure and controls are showed in Table 1.

**Table 1:** Demographic and clinical data of patients with heart failure and controls with *Pneumocystis* colonization.

**Characteristics**	**HF (*n* = 36)**	**Non-HF (*n* = 36)**	***p*-value**
Age, mean years ± SD	70.97 ± 7.5	65 ± 9.8	0.134
Female gender, %	50	50	0.813
Active smoker, %	16.6	27.7	0.395
COPD, %	13.8	0	0.063
Asthma, %	2.7	5.5	1
*Pneumocystis* colonization, %	2.7	8.3	0.606

*HF, heart failure; COPD, chronic obstructive pulmonary disease*.

When patients with HF diagnosis were compared with healthy controls, matched by age and gender, we only found *Pneumocystis* colonization in a single male patient with HF (2.78%) vs. three among healthy individuals, two men and one female (8.33%) (*p* = 0.606). None of those four colonized individuals had COPD or asthma. All of them were former smokers.

## Discussion

To the best of our knowledge, this study is the first to describe the prevalence of *Pneumocystis* colonization in patients with heart failure and to show that heart failure is not a risk factor to infection by this pathogen. Also this study is the first showing that *Pneumocystis* colonization does not seem to have a role in the pathophysiology of heart failure.

In our study, the prevalence of *Pneumocystis* colonization among patients with heart failure is similar to the prevalence in the general population, blood donors, from the same geographical area. The observed trend of lowest percentage of *Pneumocystis* colonization in patients with heart failure, compared to healthy individuals, especially when nearly 14% of heart failure patients have COPD, where *Pneumocystis* colonization is frequent (14), is not easy to explain. It could be related to lowest percentage of active smokers in heart failure patients, only 16.6% compared to 27.7% in controls, inasmuch as smoking is a risk factor for *Pneumocystis* colonization (15, 16). However, neither colonized heart failure patient nor the colonized controls were active smokers in the moment of study, although all of them were former smokers.

There are only a few studies about the prevalence of *Pneumocystis* colonization in general population. The first study developed in southern Spain found a prevalence of 20% among adults without underlying lung disease or immunosuppression (17). This prevalence is not different to the prevalence observer in our study, 8.3% (*p* = 0.136) developed in northeastern Spain and similar to the prevalence described in older adults from Chile 12.8% (*p* = 0.466) (18). In both studies, identification of *Pneumocystis* colonization was made using oropharyngeal wash specimens and the same PCR protocol. Oropharyngeal washing is preferred for epidemiological studies because of generalized good tolerance for adults and good sensitivity, which reached 95.4% compared with bronchoalveolar lavages or sputum samples in a previous study of our group (12).

This study confirms that non-immunosuppressed adults can be colonized by *Pneumocystis* and could participate in the transmission cycle of *P. jirovecii* as a source of this infection for immunosuppressed susceptible individuals, mainly in hospital environment. Results of our study about relation between *Pneumocystis* colonization and heart failure have to be interpreted with caution because the study have been made from a single center and had a small sample size. Therefore, to carry out future and more comprehensive studies to further define the role of *Pneumocysti*s infection in heart failure patients would be desirable.

## Data Availability Statement

The datasets generated for this study are available on request to the corresponding author.

## Ethics Statement

The studies involving human participants were reviewed and approved by Hospital Clínico Lozano Blesa. The patients/participants provided their written informed consent to participate in this study.

## Author Contributions

EC and MT conceived and designed the research. SM, JD, and SO collected and analyzed the data. MT and IM-C performed the statistical analysis. IM-C, VF, EC, and MT contributed to the development of the study and interpreted the data. IM-C, VF, and EC wrote the draft of the manuscript. All authors reviewed and approved the final version of the manuscript.

### Conflict of Interest

The authors declare that the research was conducted in the absence of any commercial or financial relationships that could be construed as a potential conflict of interest.
